# Serological Biomarkers for Early Detection of Hepatocellular Carcinoma: A Focus on Autoantibodies against Tumor-Associated Antigens Encoded by Cancer Driver Genes

**DOI:** 10.3390/cancers12051271

**Published:** 2020-05-18

**Authors:** Keyan Wang, Miao Li, Jiejie Qin, Guiying Sun, Liping Dai, Peng Wang, Hua Ye, Jianxiang Shi, Lin Cheng, Qian Yang, Cuipeng Qiu, Di Jiang, Xiao Wang, Jianying Zhang

**Affiliations:** 1 Department of Epidemiology and Health Statistics & Henan Key Laboratory for Tumor Epidemiology, College of Public Health, Zhengzhou University, Zhengzhou 450001, China; wangkeyan0902@163.com (K.W.); qinjie2007@126.com (J.Q.); guiyingsun18@163.com (G.S.); wangpeng@zzu.edu.cn (P.W.); yehua@zzu.edu.cn (H.Y.); qianyang727@163.com (Q.Y.); Agave25@163.com (C.Q.); 2 Henan Academy of Medical and Pharmaceutical Sciences, Zhengzhou University, Zhengzhou 450052, China; miaoli6868@163.com (M.L.); lpdai@hotmail.com (L.D.); jianxiangshi@zzu.edu.cn (J.S.); dijiang2020@hotmail.com (D.J.); 3 College of Life Science, Xinyang Normal University, Xinyang 464000, China; chenglin0630@163.com

**Keywords:** hepatocellular carcinoma (HCC), autoantibodies, biomarker, protein microarray, cancer driver gene

## Abstract

Substantial evidence manifests the occurrence of autoantibodies to tumor-associated antigens (TAAs) in the early stage of hepatocellular carcinoma (HCC), and previous studies have mainly focused on known TAAs. In the present study, protein microarrays based on cancer driver genes were customized to screen TAAs. Subsequently, autoantibodies against selected TAAs in sera were tested by enzyme-linked immunosorbent assays (ELISA) in 1175 subjects of three independent datasets (verification dataset, training dataset, and validation dataset). The verification dataset was used to verify the results from the microarrays. A logistic regression model was constructed within the training dataset; seven TAAs were included in the model and yielded an area under the receiver operating characteristic curve (AUC) of 0.831. The validation dataset further evaluated the model, exhibiting an AUC of 0.789. Remarkably, as the aggravation of HCC increased, the prediction probability (PP) of the model tended to decrease, the trend of which was contrary to alpha-fetoprotein (AFP). For AFP-negative HCC patients, the positive rate of this model reached 67.3% in the training dataset and 50.9% in the validation dataset. Screening TAAs with protein microarrays based on cancer driver genes is the latest, fast, and effective method for finding indicators of HCC. The identified anti-TAA autoantibodies can be potential biomarkers in the early detection of HCC.

## 1. Introduction

Hepatocellular carcinoma is the third leading cause of cancer death worldwide [1,2]. It was estimated that only approximately 10% of HCC patients can be detected in the early stage and is suitable for surgical resection [3,4]. This phenomenon is mainly attributed to the absence of effective early diagnostic markers [5,6,7]. Therefore, there is an urgent need to find new noninvasive biomarkers for the early detection of HCC.

Substantial evidence indicates that autoantibodies to TAAs are produced in the early stage of tumorigenesis [8,9,10]. Compared with other serological diagnostic biomarkers of HCC, such as circulating cell-free DNA [11], TAAs themselves [12], cytokines [13], or RNA [14], autoantibodies enlarged by the immune system are more stable and durable, are more easily measured, and require less blood to be drawn from the patients, making them ideal noninvasive biomarkers [15,16,17,18,19,20,21,22]. Our previous studies demonstrated that the malignant transition to HCC is associated with increased autoantibodies against certain cellular proteins and that a mini-array of eight TAAs could enhance antibody detection for the diagnosis of HCC [23]. However, the TAAs evaluated previously were mostly achieved from scattered studies or reports on other types of cancer. Thus, it is necessary to identify novel representative TAAs for the detection of HCC.

The T7-phase display system [24], recombinant cDNA expression library (SEREX) [25], and Serological Proteome Analysis (SERPA) [26,27,28] have been performed in previous studies to screen TAAs for the diagnosis of HCC. However, these methods not only have tedious steps and strict technical requirements for operators but also have relatively high false positive rates. Therefore, a growing number of studies focused on protein microarrays to screen TAAs in a single experiment and have facilitated the rapid and efficient identification of novel biomarkers [29,30]. Hepatocarcinogenesis is a complex multistep process involving many altered signaling cascades, and the most important change in this process is the mutation of cancer driver genes [31]. It has been hypothesized that the proteins encoded by cancer driver genes might be changed following cancer driver genes’ mutation; accordingly, the corresponding autoantibodies might be produced or have their levels increased. Therefore, in the current study, a customized protein microarray based on cancer driver genes was applied to screen TAAs with sera from 100 HCC patients and 50 normal controls (NCs). After verification with ELISA in the same set of samples, another 898 samples, divided into a training dataset and validation dataset, were also tested by ELISA to evaluate the potential of anti-TAA autoantibodies as noninvasive biomarkers for the detection of HCC.

## 2. Results

### 2.1. Serum Samples and Study Design

**Figure 1:**
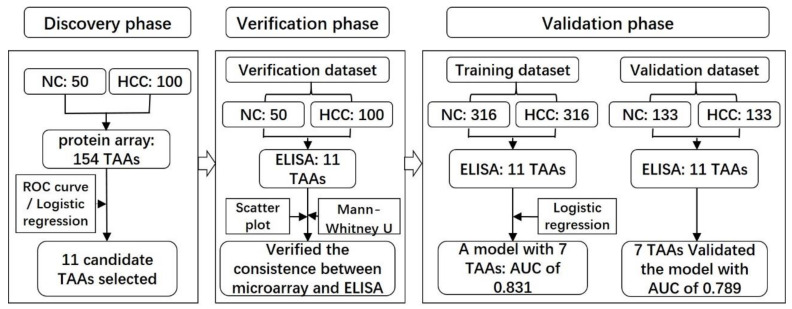
Workflow chart of the study. A total of 150 samples were used to screen potential biomarkers in the discovery phase and further applied in the verification dataset to verify the results of the protein microarrays. Another 898 participants were included in the validation phase, 632 of which were used to validate the previous results and to build a predictive model for HCC named as the training dataset, while the other 266 participants were recruited to validate the model named as the validation dataset. HCC, hepatocellular carcinoma; NC, normal control.

In the initial study, 100 HCC sera and 50 NC sera used in the discovery phase and verification phase were randomly selected from the serum bank of Tumor Epidemiology Laboratory of Zhengzhou University (Henan, China). In the subsequent validation phase, all serum samples, including 127 liver cirrhosis (LC) sera and 449 HCC sera as well as 449 NC sera matched to HCC patients by age and gender, were obtained from the same serum bank of Tumor Epidemiology Laboratory of Zhengzhou University. These serum samples were independently classified into the training dataset and validation dataset according to the collection time of patients and tested by ELISA, and the detailed information is illustrated below in Figure 1. Written informed consent was obtained from all participants. All normal controls had no evidence of cancer history or hepatic diseases. All HCC patients were diagnosed following the Chinese Guidelines for Diagnosis of HCC (version 2017) [32]. Tumor stage was defined according to the eighth edition of the American Joint Committee on Cancer (AJCC) Cancer Staging Manual [33]. This study was approved by the Ethics Committee of Zhengzhou University (ZZURIB 2019-001).

The demographic and clinical characteristics of the participants were analyzed. The distributions of the TNM stage, metastasis status, gender, and age were not significantly different between the training and validation datasets (all *p* > 0.05). However, gender and age in the verification dataset were not completely matched between HCC patients and NCs. The positive rate of AFP was less than 60% for HCC patients in each dataset, while it was 40.9% for patients with liver cirrhosis, as shown in Table 1.

### 2.2. Serum Autoantibody in Discovery and Verification Phase 

Higher titers of autoantibodies against 128 TAAs were observed in HCC patients than those in NCs (AUC > 0.5, *p* < 0.05), among which 4 targets (GNA11, PTCH1, PAX5, SRSF2) were screened by logistic regression with a combined AUC of 0.825. In addition, 20 markers with higher positive rates in HCC patients than in NCs, when the cutoff values were set as the mean + 2SD, mean + 3SD, and 95% quantile of NCs, respectively, were also included in logistic regression. As a result, three TAAs (GNA11, MSH2, and GNAS) with a combined AUC of 0.767 were selected. Moreover, IDH1, PTEN, NPM1, survivin, and TP53 presented higher AUCs (0.710, 0.690, 0.690, 0.690, and 0.630, respectively) than others. Taken together, 11 TAAs were included in the subsequent study, as shown in Figure S2.

**Figure 2:**
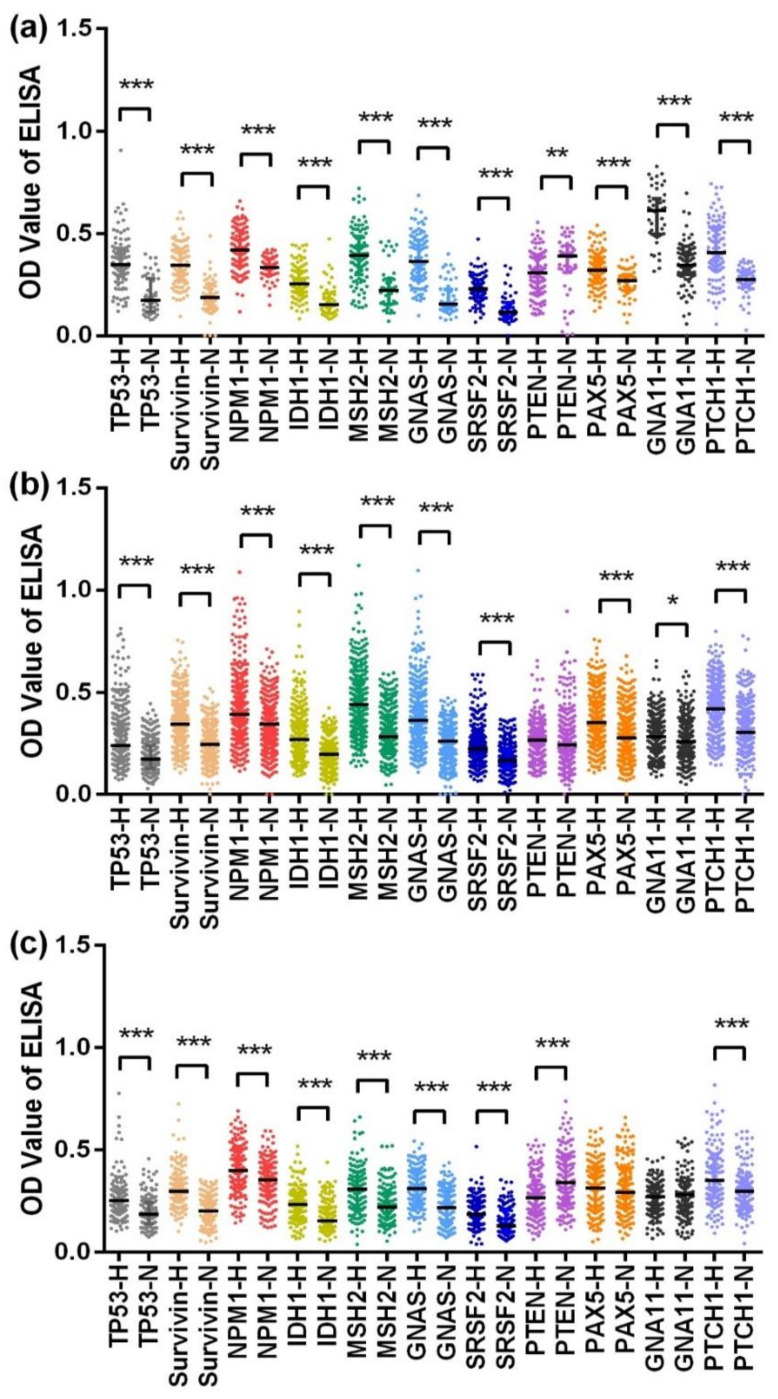
Scatter plots of the optical density (OD) value of ELISA for 11 anti-TAA autoantibodies in the verification dataset (**a**), training dataset (**b**), and validation dataset (**c**). *** *p* < 0.001, ** *p* < 0.01, * *p* < 0.05. H, hepatocellular carcinoma; N, normal control. The lines on scatter plots are median with interquartile range.

The results from the verification phase were consistent with those from the discovery phase except the autoantibody against PTEN. The level of anti-PTEN autoantibody was significantly higher in the NCs than that in the HCC patients, as shown in Figure 2a.

### 2.3. Serum Autoantibody in the Validation Phase

In the training dataset, the levels of 11 anti-TAA autoantibodies were significantly higher in the HCC patients than in the normal controls, except for those of the anti-PTEN autoantibody, for which there was no difference between the two groups, as shown in Figure 2b. In the validation dataset, anti-PAX5 and anti-GNA11 autoantibodies showed little difference in their levels between the HCC patients and NCs, and the level of anti-PTEN autoantibody was lower in the HCC patients than in the NCs, while the other indicators all presented higher levels in the HCC patients than in the NCs, as shown in Figure 2c.

Receiver operator characteristic (ROC) curve analysis was performed to evaluate the diagnostic value of each anti-TAA autoantibody in the training dataset, as shown in Figure S3. The AUCs ranged from 0.545 to 0.766, and the sensitivities were distributed in the range of 3.8–48.4%, and when specificities were maintained above 90%, the points with maximum sensitivity were set as the cutoff values. Autoantibodies against MSH2, GNAS, and survivin presented the highest diagnostic performance for HCC, with AUCs of 0.766, 0.747, and 0.750, and sensitivities of 42.1%, 48.4%, and 42.4%, respectively (all *p* < 0.001).

**Figure 3:**
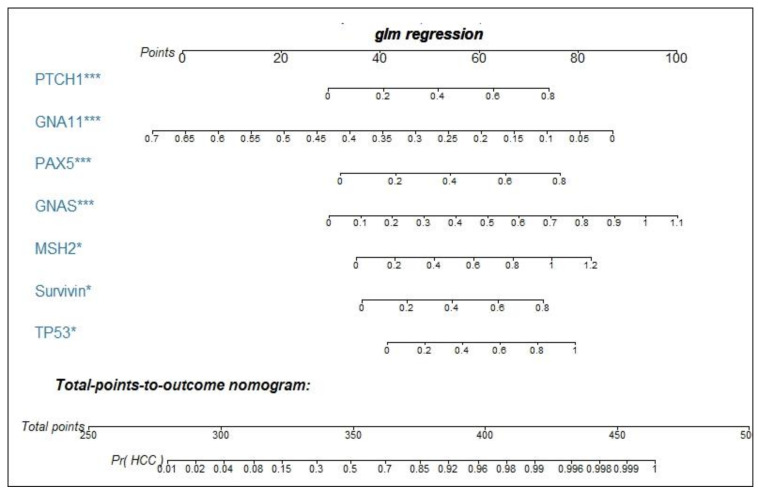
Nomogram for predicting the probability of HCC. As an example, locate the patient’s OD value of anti-PTCH autoantibody and draw a line straight up to the “points” axis to determine the score associated with this indicator. Add the scores achieved for each anti-TAA autoantibody, and locate this sum on the “total points” axis. Draw a line straight down to determine the probability of HCC. *** *p* < 0.001, * *p* < 0.05. Pr (HCC), probability of HCC.

**Figure 4:**
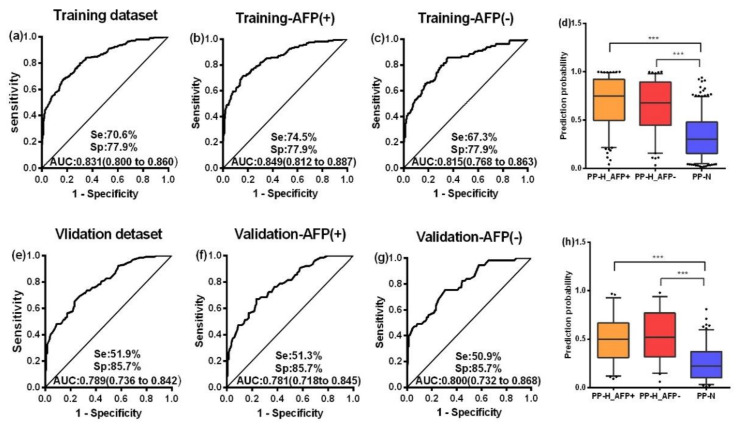
ROC curves of the PP value in the training dataset (**a**), validation dataset (**e**), AFP (+) group and AFP (−) group of the training dataset (**b**,**c**), and those of the validation dataset (**f**,**g**). Box plots present the levels of the PP value among the AFP (+) group and AFP (−) group and NC group in the training dataset (**d**) and validation dataset (**h**). Se, sensitivity; Sp, specificity; AUC, area under the receiver operating characteristic curve, 95%CI of AUC in brackets. PP, the prediction probability of logistic regression for HCC; H, hepatocellular carcinoma. Lines on boxes are in the order of 95, 75, 50, 25 and 5 quantiles from top to bottom; Whiskers indicate points greater than 95% quantile and less than 5% quantile; *** *p* < 0.001.

Binary logistic regression was performed by using the training dataset to build a predictive model for HCC. When the “Forward: conditional” method was used, seven markers were included in the model with the following equation: Prediction probability value of HCC = 1 / (1 + Exp (−(2.522 × TP53 + 3.042 × Survivin + 2.625 × MSH2 + 4.241 × GNAS + 3.676 × PAX5 − 8.789 × GNA11 + 3.688 × PTCH1 − 3.863))). A nomogram was produced according to the logistic regression model, as shown in Figure 3. The cutoff value used to differentiate HCC patients from NCs was set as 0.5. This model yielded an AUC of 0.831 and a consistency rate of 74.2% in the training dataset, as shown in Figure 4a and Table 2. Then, the validation dataset was applied to validate the model, and a slightly lower AUC that was not statistically different from that in the training dataset (*z* = 1.338, *p* = 0.1808) was obtained, as shown in Figure 4e and Table 2.

The ROC curves of PP produced by the logistic regression were analyzed and compared between the early stage (AJCC, stage I–II) and late stage (stage III–IV) of HCC. The AUCs of PP in the training dataset and the validation dataset of the early stage were 0.837 and 0.839, while those of the late stage were 0.835 and 0.774, respectively. No significant difference was discovered for the diagnostic performance of the model between the early stage and late stage of HCC in either the training dataset or the validation dataset (all *p* > 0.05), as shown in Figure S4 and Table S1.

### 2.4. AFP and Autoantibodies

According to the cutoff value of AFP (20 ng/mL), HCC patients were divided into an AFP-positive group (AFP+) and an AFP-negative group (AFP-). The ROC curves of the PP value were generated in the two groups both in the training dataset and validation dataset, as shown in Figure 4b,c,f,g. No difference was seen in the AUCs between the two groups in either the training or the validation dataset (all *p* > 0.05). Similarly, the positive rates and levels of the PP value between the AFP (+) group and AFP (−) group were not different in either the training or the validation datasets, as shown in Table S2 and Figure 4d,h. Based on the above findings, the performance of AFP and the PP value to diagnose HCC independently and in combination was further analyzed. There was no significant difference between AFP and the PP value in differentiating HCC patients from NCs whether in the training dataset (*z* = 1.242, *p* = 0.214) or in the validation dataset (*z* = 0.268, *p* = 0.789), but the sensitivity in the early stage of HCC predicted by the PP value was higher than that predicted by AFP (*p* = 0.019), as shown in Figure S5. When AFP and the PP value were combined by logistic regression to classify HCC patients and NCs, multiple evaluating indicators, especially, sensitivity, AUC, the Youden’s index, accuracy, and the kappa value, significantly increased with respect to those by AFP or PP alone in both the training and validation datasets, as shown in Table 2 and Figure S5. 

**Figure 5:**
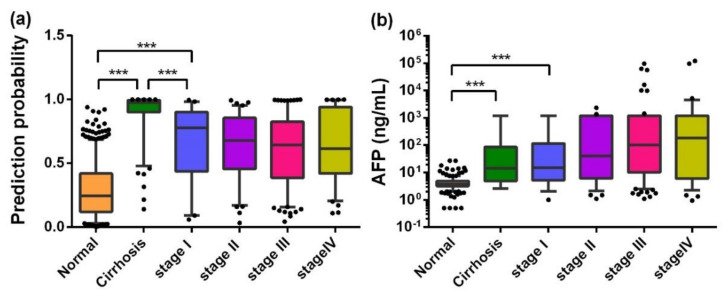
Box plots of PP (**a**) and AFP (**b**) in different stages in the development of HCC. Lines on the boxes are in the order of 95, 75, 50, 25, and 5 quantiles from top to bottom; Whiskers indicate points greater than the 95% quantile and less than 5% quantile; *** *p* < 0.001.

When the training dataset and the validation dataset were combined and 127 liver cirrhosis cases were included as precancerous HCC lesions, the trends of AFP and the PP value as biomarkers in the progression of HCC were analyzed. Interestingly, as HCC developed and its aggressiveness increased, the AFP value tended to increase (median: NC of 3.80, LC of 14.32, stage I of 14.88, stage II of 39.80, stage III of 100.12, stage IV of 179.21), while the PP value tended to decrease a little (median: NC of 0.246, LC of 0.976, stage I of 0.780, stage II of 0.679, stage III of 0.643, stage IV of 0.615). Most worthy of attention was the difference among the PP values for NCs, liver cirrhosis, and stage I HCC patients, as shown in Figure 5. Additionally, the PP values of HCC patients with metastasis appeared lower than those without metastasis, and those of the HCC patients with high-grade pathological differentiation were lower than others, but none of these differences were significant, as shown in Figure S6.

## 3. Discussion

There are several novel features in the current study. (1) A new modality to screen TAAs with protein microarrays based on cancer driver genes was utilized for the detection of HCC. (2) Multiple phases of validation were conducted in a large sample of patients and controls. (3) A parallel comparison between TAAs and AFP was performed and led to the conclusion that anti-TAA autoantibodies are produced earlier in sera than AFP, indicating that a panel of anti-TAA autoantibodies could be promising biomarkers in the early detection of HCC.

In the discovery phase, 11 candidate TAAs were selected. Their function and detailed description are summarized in Table S3 [34,35,36,37,38,39,40,41,42,43,44]. Autoantibodies to GNA11, GNAS, PTCH1, PAX5, MSH2, IDH1, and SRSF2 were first reported and applied for the detection of HCC. The remaining four TAAs (PTEN, TP53, NPM1, and survivin) and their autoantibodies have been studied previously in multiple cancers [23,45,46,47,48]. We believe that we are the first to perform the initial screening of TAAs through customized protein microarrays with high-throughput screening targets based on cancer driver genes. These protein microarrays can not only overcome tedious work and require minute amounts of patient sera but also have a higher input-output ratio than whole human proteome arrays.

In the verification phase, 10 out of the 11 anti-TAA autoantibodies presented similar results to those of the protein microarray. These results have not only verified the reliability of the protein microarray but also highlighted the consistency of ELISA and protein microarray, which paved the way for the subsequent validation of a large population by ELISA and other immunoassays.

In the validation phase, GNAS, MSH2, and survivin displayed better diagnostic performances than other TAAs, while PTEN was not a good performer, which was consistent with previous verification results, and may have been caused by the reduction of PTEN expression in HCC tissue [49,50]. Although autoantibodies against PAX5 and GNA11 showed little difference between the HCC and NC groups in the process of univariate analysis in the validation dataset, they were still selected as statistically significant predictors (risk factors) for HCC in multivariate analysis by logistic regression in the training dataset. Since this process can also adjust the confounding factors, therefore, these two indicators were also included in the model. Overall, the sensitivity of a single autoantibody was low, which is consistent with most studies in the field of TAA [16,19,23,47]. This phenomenon may be attributed to the fact that tumorigenesis is a multistep process involving several genes and affected by a variety of environmental factors. To circumvent this weakness, a binary logistic regression was employed to construct a predictive model for HCC. Seven biomarkers (MSH2, GNAS, PAX5, GNA11, PTCH1, TP53, survivin) were incorporated into the model with an AUC of 0.831. From this model, it was shown that GNAS exhibited the highest odds ratio of 69.46, followed by PTCH1 with an odds ratio of 39.95. It was concluded that GNAS and PTCH1 may play more important roles in the prediction of HCC than the other indicators. One of our previous studies used 14 TAAs to diagnose HCC in parallel, resulting in a sensitivity of 69.7%, and a slightly higher specificity of 83.0% in 165 participants [27]. Parallel detection of 10 TAAs in another study in 159 samples to diagnose HCC obtained a specificity of 87.8% but a sensitivity of 66.0% [51]. Middleton et al. found that a 21-TAA panel achieved a specificity of 92% and sensitivity of 45% for the detection of HCC in 57 HCC patients and 169 controls [52]. None of these studies with small sample sizes established a predictive model for HCC, let alone performed a subsequent validation.

When HCC patients were divided into the early stage and late stage, no significant differences of the PP value were found. The results agreed with the studies of Wang et al. in gastric cancer [47] and Zhang et al. in esophageal squamous cell carcinoma [53]. This result indicated that autoantibodies against TAAs might change little after cancer is diagnosed.

AFP has generally been used as a serological marker for the diagnosis of HCC in clinical practice. One of the focuses of this study was to determine the association, if any, between AFP and TAAs in the detection of HCC. After comparing the AUC value of the model in the AFP-positive and AFP-negative groups, it was concluded that anti-TAA autoantibodies were not associated with AFP. In other words, anti-TAA autoantibodies were independent markers, and they could be used in the subjects with negative AFP values to detect HCC. We attempted to combine AFP and the PP value to predict HCC, obtaining better results in the training dataset and validation dataset that were significantly higher than those of either AFP or the TAAs alone (*p* < 0.05). This result was in line with the findings in Wang’s study [46], which focused on only three autoantibodies from studies with a small sample size, and concluded that autoantibodies may be useful biomarkers in the diagnosis of AFP-negative HCC patients. Similarly, in another study by Yu et al. [26], it was concluded that autoantibodies to TAAs may be complementary to AFP and could improve the diagnosis of early HCC. 

Interestingly, the PP value produced by the logistic regression model was higher for stage I HCC than for the other stages, which was fully understood when including liver cirrhosis as a premalignant lesion (Figure 5). Different trends between AFP and the PP value were observed in the progression of HCC. Moreover, the positive rate in the early stage of HCC predicted by the PP was higher than that predicted by AFP (Figure S5). These phenomena further illustrated that the PP value and AFP are two independent markers and can complement each other in the detection of HCC. Additionally, although the mechanism of autoantibody production warrants further study, it was speculated that autoantibodies to TAAs may be produced very early in the development of HCC, before being detected by any medical imaging examination. Hence, anti-TAA autoantibodies can be promising serological markers for the early screening of HCC, which can greatly reduce the social and family burden. However, one limitation of the current study was that we failed to track the outcome of patients with liver cirrhosis, and their successive serum samples were not available. This will be addressed in our future research.

## 4. Materials and Methods 

### 4.1. Expression and Purification of Recombinant TAAs

The proteins on microarrays were expressed in yeast and affinity purified (CDI Laboratories Inc, Mayaguez, PR, USA). The proteins used in ELISA were obtained as follow: TP53, NPM1, and survivin were expressed and purified in our laboratory as described previously [27,54]; GNA11, PTCH1, PTEN, and PAX5 were purchased from LD Biopharma Inc (San Diego, CA, USA); GNAS, MSH2, IDH1, and SRSF2 were bought from cloud-clone corporation (Wuhan, China). The purity, concentration, and molecular weight of each protein were verified by SDS-PAGE before being used in ELISA.

### 4.2. Human Protein Microarray Assay

A total of 154 proteins or protein fragments were arranged in a microarray, 143 of which were encoded by cancer driver genes reported by Vogelstein et al. [31]; the other 11 proteins (CIP2A/p90, RalA, IMP1, IMP2, IMP3, CyclinB1, c-Myc, YWHAZ, RBM39, and two fragments of survivin) previously studied in our research group were also included. Full-length proteins encoded by 18 genes were not obtained, so more than two fragments of these proteins were used instead, as shown in Figure S1.

Autoantibodies in sera were combined with the proteins coated on the microarrays, the unbound ones were washed away, and the remaining ones were then reacted with anti-human IgG fluorescent secondary antibodies. Finally, a fluorescent scanner was implemented to read the signal. Additional details can be found in a previous study [55]. For data readout, three of the sera from the HCC patients were not taken into account because of their high levels of background signal. Moreover, to minimize the deviation caused by the inconsistent background signal, the ratio of the foreground to the background intensities (SNR: signal to noise ratio) of each protein was used in the following analysis.

### 4.3. Enzyme-Linked Immunosorbent Assay (ELISA) 

Autoantibodies against 11 TAAs were detected by ELISA. The 11 purified recombinant proteins were diluted in coating buffer at optimal concentrations (TP53, survivin, MSH2, GNAS, PTEN, PAX5, GNA11, PTCH1, IDH1, SRSF2, and NPM1 at final concentrations of 0.5, 0.5, 0.25, 0.25, 0.25, 0.25, 0.5, 0.5, 0.125, 0.125, and 0.125 µg/mL, respectively). The other details were described previously [20,47]. All cancer and normal samples were interspersed on the plates, and 8 fixed human serum samples and 2 blank controls were run on each plate. The mean value of the 8 samples was used to normalize the different plates, and the blank controls were consulted for quality control for individual plates.

### 4.4. Statistical Analysis

Data analysis was performed by SPSS (Version 21.0, Chicago, IL, USA) and GraphPad Prism software (version 6.0, La Jolla, CA, USA). The nomogram was generated by R3.6.1. The Chi-squared test or Student’s *t* test was applied to compare the differences in characteristics between the two groups. The Mann–Whitney U test was performed when comparing SNR values or OD values between HCC patients and NCs. Differences between the two subgroups were examined by the Kruskal–Wallis test. Logistic regression was employed to screen risk factors for HCC in the discovery phase and to establish a predictive model for HCC in the validation phase. ROC curves were generated, and the AUC was used to evaluate the diagnostic value of each anti-TAA autoantibody. In all tests, a *p* value of <0.05 (two sided) was considered to be significant.

## 5. Conclusions

In summary, using a novel screening method based on a large cohort of subjects, a panel of seven anti-TAA autoantibodies as biomarkers were identified and validated, which can provide a far-reaching approach in the detection of HCC, especially for AFP-negative HCC. Additionally, we note that the sensitivity predicted by the PP value in the early stage of HCC was higher than that predicted by AFP, and the PP value tended to decrease from LC to HCC of stage I then to HCC of stage II, which suggests that anti-TAA autoantibodies might occur very early in the development of HCC and may be used as serological biomarkers in the early screening of HCC.

## Figures and Tables

**Table 1:** Characteristics of participants.

Characteristics	Verification Dataset		Training Dataset		Validation Dataset		LC (*n* = 127)
	HCC (*n* = 100)	NC (*n* = 50)	HCC (*n* = 316)	NC (*n* = 316)	HCC (*n* = 133)	NC (*n* = 133)	
Male, *n* (%)	79 (81.4)	23 (46.0)	260 (82.3)	245 (77.5)	98 (73.7)	96 (72.2)	87 (68.5)
Age							
Mean ± SD	40.5 ± 13.0	56.8 ± 9.3	55.4 ± 12.5	56.7 ± 11.4	55.9 ± 10.0	56.1 ± 10.0	52.0 ± 11.1
Age range	37.0−77.5	20.0−71.0	24.0−87.0	32.5−88.0	26.0−79.0	34.0−80.0	24.0−78.0
TNM, *n* (%)							
I	35 (36.1)	IA	28 (8.9)	IA	13 (9.8)	IA	IA
II	19 (19.6)	IA	63 (19.9)	IA	21 (15.8)	IA	IA
III	26 (26.8)	IA	129 (40.8)	IA	62 (46.6)	IA	IA
IV	17 (17.5)	IA	60 (19.0)	IA	22 (16.5)	IA	IA
NA	0 (0)	IA	36 (11.4)	IA	15 (11.3)	IA	IA
Metastasis, *n* (%)							
None	83 (85.6)	IA	263 (83.2)	IA	112 (84.2)	IA	IA
With	14 (14.4)	IA	53 (16.8)	IA	21 (15.8)	IA	IA
AFP, *n* (%)							
≥20 ng/mL	57 (58.8)	0 (0)	161 (50.9)	2 (0.6)	76 (57.1)	0 (0)	52 (40.9)
<20 ng/mL	40 (41.2)	50 (100)	113 (35.8)	259 (82.0)	57 (42.9)	73 (54.9)	75 (59.1)
NA	0 (0)	0 (0)	42 (13.3)	55 (17.4)	0 (0)	60 (45.1)	0 (0)

HCC, hepatocellular carcinoma; NC, normal control; LC, liver cirrhosis; SD, standard deviation; AFP, alpha-fetoprotein; NA, not available; IA, Inapplicable.

**Table 2:** The diagnostic value of PP, AFP, and their combination in the training dataset and validation dataset.

Markers	AUC (95%CI)	Se (%)	Sp (%)	Youden’s Index	PLR	NLR	PPV (%)	NPV (%)	Accuracy (%)	Kappa
Training dataset										
PP	0.831 (0.800, 0.860)	70.6	77.8	0.484	3.186	0.378	76.1	72.6	74.2	0.484
AFP	0.860 (0.826, 0.894)	58.8	99.2	0.580	76.681	0.416	98.8	69.6	78.5	0.574
PP + AFP	0.929 (0.908, 0.951)	81.0	92.3	0.734	10.573	0.206	91.7	82.3	86.5	0.731
Validation dataset										
PP	0.789 (0.736, 0.842)	51.9	85.7	0.376	3.632	0.561	78.4	64.0	68.8	0.376
AFP	0.800 (0.738, 0.861)	57.1	100	0.571	+ ∞	0.429	100	56.2	72.3	0.486
PP + AFP	0.891 (0.847, 0.934)	74.4	97.3	0.717	27.169	0.263	98.0	67.6	82.5	0.652

PP, prediction probability of logistic regression; AFP, alpha-fetoprotein; Se, sensitivity; Sp, specificity; AUC, area under the receiver operating characteristic curve; CI, confidence interval; PLR, positive likelihood ratio; NLR, negative likelihood ratio; PPV, positive predictive value; NPV, negative predictive value; + ∞, positive infinity.

## Supplementary Materials

The following are available online at https://www.mdpi.com/2072-6694/12/5/1271/s1, Figure S1: Layout of recombinant proteins on customized proteome microarrays, Figure S2: Scatter plots and ROC curves of autoantibodies against 11 selected TAAs from protein microarray, Figure S3: ROC curves of single anti-TAA autoantibody in training dataset, Figure S4: ROC curves of prediction probability value in early stage and late stage of training dataset (**a**,**b**) and validation dataset (**c**,**d**), Figure S5: Positive rate of PP, AFP and PP&AFP in NC, cirrhosis, stage I-II HCC patients and stage III-IV HCC patients, Figure S6: Box plots of PP value and AFP in different histological grades (**a**,**b**) and metastasis (**c**,**d**) of HCC, Table S1: Positive rate in different stage of different datasets, Table S2: Positive rate in AFP (+) and AFP (−) group of different datasets, Table S3: Descriptions/functions of the 11 TAAs.
