# Supplementary material for: Serological Biomarkers for Early Detection of Hepatocellular Carcinoma: A Focus on Autoantibodies against Tumor-Associated Antigens Encoded by Cancer Driver Genes
Source: Cancers (Basel). 2020 May 18;12(5):1271. doi: 10.3390/cancers12051271 (PMC7280966; doi:10.3390/cancers12051271)
Supplement: Supplementary file 1 [file cancers-12-01271-s001.pdf]

cancers

Supplementary Materials: Serological Biomarkers for Early Detection of Hepatocellular Carcinoma: A Focus on Autoantibodies against Tumor-Associated Antigens Encoded by Cancer Driver Genes

PTPN11	KRAS	NRAS	CCND1	PBRM1（frag)	IKZF1	Survivin	PHF6	IMP2/p62	FUBP1
NFE2L2	IMP1	U2AF1	ATRX	HRAS	AKT1	Survivin	MYCL1	IMP3/Koc	NPM1
KLF4	H3F3A	IDH2	GATA3	TP53	GNAS	NF2	SMAD2	MEN1	GATA1
PPP2R1A	WT1	PTCH1	DNMT3A	RalA	GATA3	PIK3R1	SMAD4	B2M	CTNNB1
SMARCB1	GNAQ	FGFR2	BAP1	SMO	TRAF7	SOX9	RET	GNAS	ACVR1B
SETBP1	MDM4	ASXL1	NF2	CASP8	PAX5	MPL	FBXW7	DAXX	PIK3R1
TET2（frag)	SKP2	DNMT3A	MDM2	KRAS	PRDM1	FOXL2	MAP2K4	MDM2	STAG2
GATA2	MYCN	BRCA1	MLH1	BCL2	ATM	CDC73	VHL	CDKN2C	GATA1
CBL	HRAS	CDH1	EGFR	PDGFRA	SRSF2	PTEN	MDM2	SF3B1	LMO1
BCOR	GNA11	PTEN	SRSF2	PRDM1	EGFR	IDH1	TNFAIP3	YWHAZ	KIT
CASP8	HNF1A	KLF4	NCOA3	KDM5C	SMARCA4	EZH2	RBM39	PDGFRA	NPM1
JAK2	PIK3CA	FGFR2	FGFR2	PDGFRA	RNF43	KDM6A	JAK3	BRAF	CSF1R
JAK1	ALK	CIP2A/p90	MSH6	c-Myc	APC	HIST1H3B	SOCS1	ERBB2	TSHR
ARID2	CRLF2	FGFR3	TSC1	MED12	MET	CDKN2A	MSH2	SPOP	RUNX1
Cyclin B1	RB1	NKX2-1	PRDM1	MYD88	DNMT3A	PTCH1	CEBPA	GNAS	BRCA2
NCOR1	GNAS	EGFR	ERBB2	positive3-1	positive3-2	positive3-3	positive3-4	buffer	buffer

Figure S1. Layout of recombinant proteins on customized protein microarrays. A microarray was composed of 16 rows and 20 columns, each protein was repeatedly coated on two adjacent locations in the same row, so were the control probes. No duplicate proteins were listed in the above arrangement.

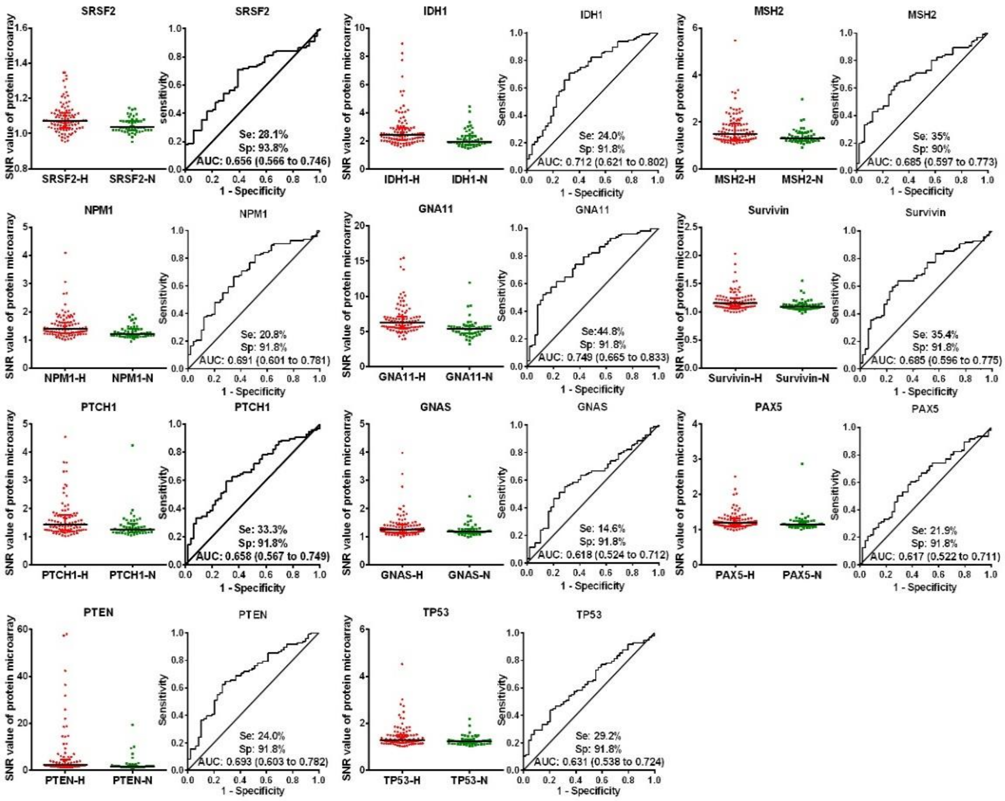

Figure S2. Scatter plots and ROC curves of autoantibodies against 11 selected TAAs from protein microarray. For each autoantibody against TAA, scatter plots of SNR value of protein microarray are on the left, while ROC curves are on the right. H, hepatocellular carcinoma; N, normal control; Se, sensitivity; Sp, specificity; AUC, area under the receiver operating characteristic curve, and 95% CI of AUC in brackets. The lines on scatter plots are median with interquartile range.

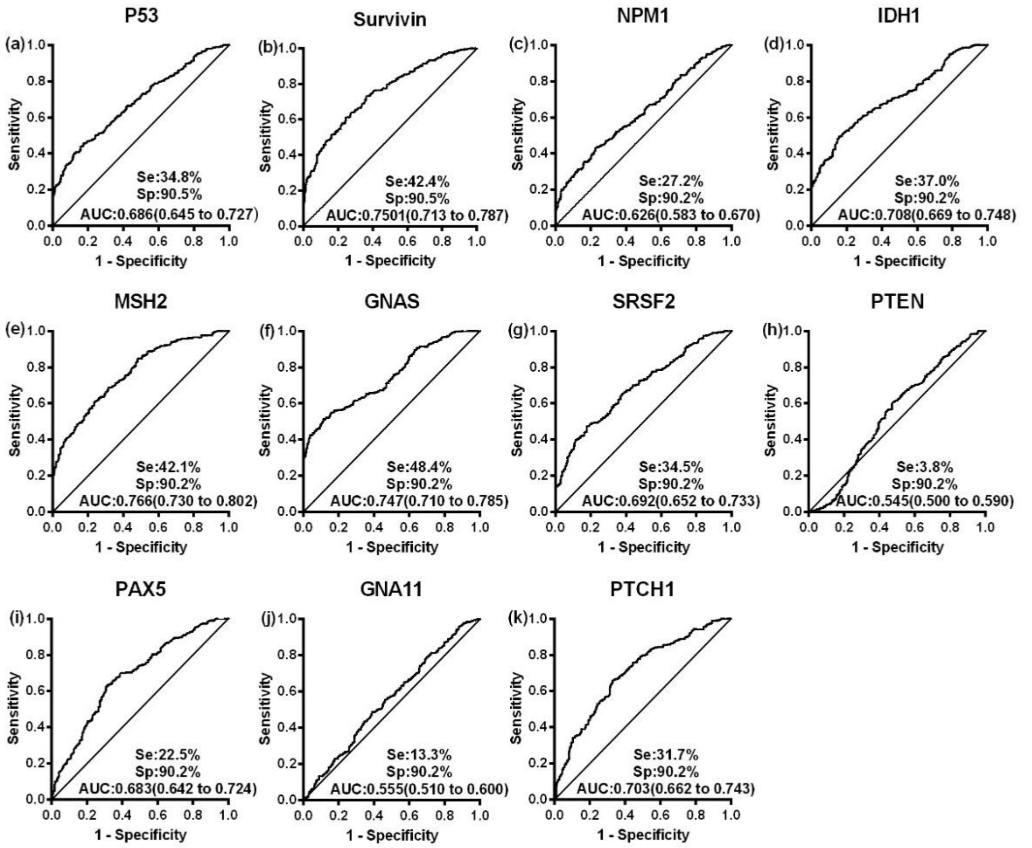

Figure S3. ROC curves of single anti-TAA autoantibody in training dataset. Se, sensitivity; Sp, specificity; AUC, area under the receiver operating characteristic curve, and 95% CI of AUC in brackets.

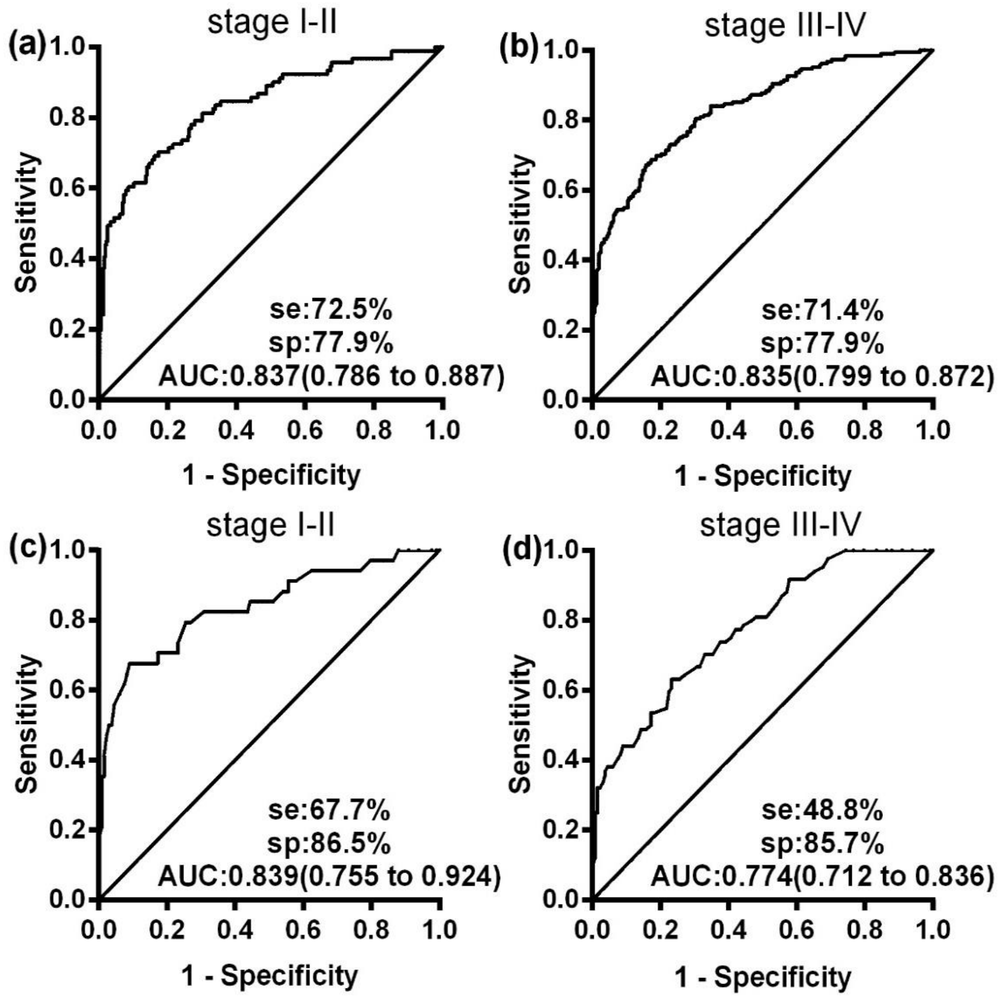

Figure S4. ROC curves of prediction probability value in early stage and late stage of training dataset (**a**,**b**) and validation dataset (**c**,**d**). Se, sensitivity; Sp, specificity; AUC, area under the receiver operating characteristic curve, and 95% CI of AUC in brackets.

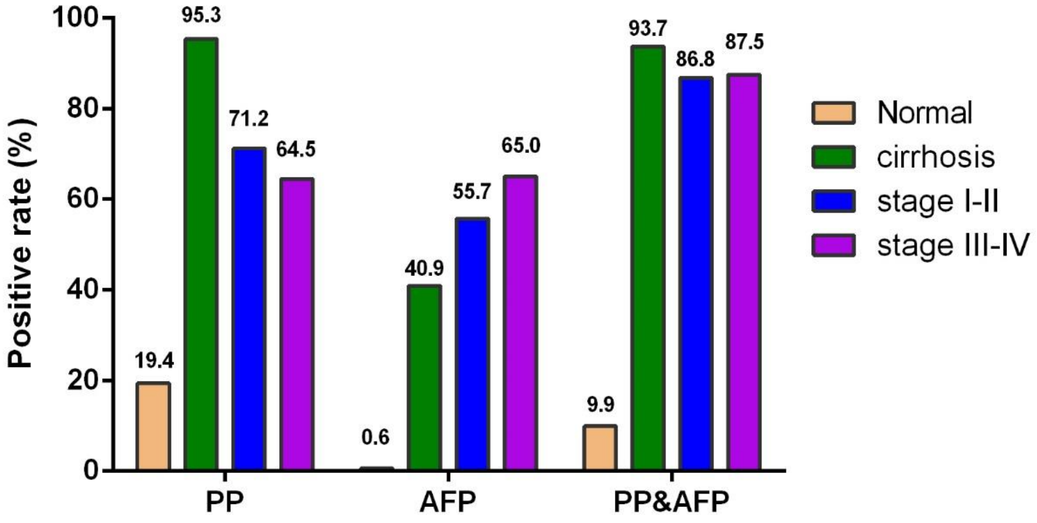

Figure S5. Positive rate of PP, AFP and PP&AFP in NC, cirrhosis, stage I-II HCC patients and stage III-IV HCC patients. PP, prediction probability; PP&AFP, PP and AFP value combined by logistic regression.

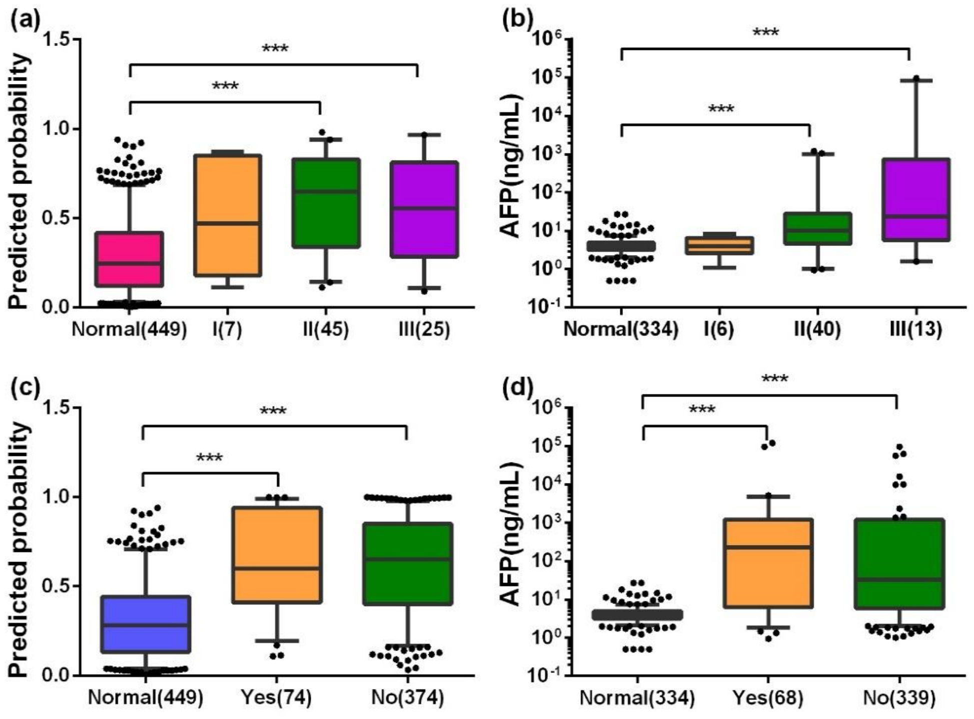

Figure S6. Box plots of PP value and AFP in different histological grades (**a**,**b**) and metastasis (**c**,**d**) of HCC. "I, II, III" indicate the grade of pathological differentiation "high, moderate, low". "Yes" and "No" represent "with metastasis" and "without metastasis" respectively. *** $P < 0.001$. Lines on boxes are in the order of 95, 75, 50, 25 and 5 quantiles from top to bottom. Whiskers indicate points greater than 95% quantile and less than 5% quantile.

Table S1. positive rate in different stage of different datasets

		Positive Rate	χ^2 / *P*
training dataset	I-II	72.5% (66 / 91)	0.645 / 0.493
	III-IV	71.4% (135 / 189)	
validation dataset	I-II	67.7% (23 / 34)	3.460 / 0.070
	III-IV	48.8% (41 / 84)	

Table S2. positive rate in AFP (+) and AFP (-) group of different datasets

		Positive Rate	χ^2 / *P*
training dataset	AFP (+)	74.5% (120 / 161)	1.727/0.221
	AFP (-)	67.3% (76 / 113)	
validation dataset	AFP (+)	51.3% (39 / 76)	0.003/1.000
	AFP (-)	50.9% (29 / 57)	

Table S3. Descriptions/functions of the 11 TAAs

TAAs	Gene Name	Classification	Core Pathway	Process
GNA11	Guanine nucleotide-binding protein subunit alpha-11	Oncogene	PI3K; RAS; MAPK	Cell Survival
MSH2	mutS homolog 2	TSG	DNA Damage Control	Genome Maintenance
GNAS	GNAS complex locus	Oncogene	APC; PI3K; TGF-b, RAS	Cell Survival / Cell Fate
PTCH1	patched homolog 1	TSG	HH	Cell Fate
IDH1	isocitrate dehydrogenase 1 (NADP+), soluble	Oncogene	Chromatin Modification	Cell Fate
SRSF2	SRSF2 serine/arginine-rich splicing factor 2	Oncogene	Transcriptional Regulation	Cell Fate
PAX5	paired box 5	TSG	Chromatin Modification	Cell Fate
Survivin / IAP4	BIRC5	------	Inhibitor of apoptosis	Apoptosis
NPM1	nucleophosmin 1 (nucleolar phosphoprotein B23, numatrin) pseudogene 21; hypothetical LOC100131044; similar to nucleophosmin 1; nucleophosmin (nucleolar phosphoprotein B23, numatrin)	TSG	Cell Cycle / Apoptosis	Cell Survival
TP53	tumor protein p53	TSG	Cell Cycle / Apoptosis; DNA Damage Control	Cell Survival
PTEN	Phosphatidylinositol 3,4,5-trisphosphate 3-phosphatase and dual-specificity protein phosphatase	TSG	PI3K	Cell Survival

TSG: tumor suppressor gene.
